# Using protein geometry to optimize cytotoxicity and the cytokine window of a ROR1 specific T cell engager

**DOI:** 10.3389/fimmu.2024.1323049

**Published:** 2024-02-22

**Authors:** Xueyuan Zhou, Felix Klaus Geyer, Dominic Happel, Jeffrey Takimoto, Harald Kolmar, Brian Rabinovich

**Affiliations:** ^1^Drug Discovery and Development, Fuse Biotherapeutics, Woburn, MA, United States; ^2^Institute for Organic Chemistry and Biochemistry, Technical University of Darmstadt, Darmstadt, Germany; ^3^Centre for Synthetic Biology, Technical University of Darmstadt, Darmstadt, Germany

**Keywords:** T cell engager, cytokine release syndrome, ROR1, decoupling of cytotoxicity, VHH

## Abstract

T cell engaging bispecific antibodies have shown clinical proof of concept for hematologic malignancies. Still, cytokine release syndrome, neurotoxicity, and on-target-off-tumor toxicity, especially in the solid tumor setting, represent major obstacles. Second generation TCEs have been described that decouple cytotoxicity from cytokine release by reducing the apparent binding affinity for CD3 and/or the TAA but the results of such engineering have generally led only to reduced maximum induction of cytokine release and often at the expense of maximum cytotoxicity. Using ROR1 as our model TAA and highly modular camelid nanobodies, we describe the engineering of a next generation decoupled TCE that incorporates a “cytokine window” defined as a dose range in which maximal killing is reached but cytokine release may be modulated from very low for safety to nearly that induced by first generation TCEs. This latter attribute supports pro-inflammatory anti-tumor activity including bystander killing and can potentially be used by clinicians to safely titrate patient dose to that which mediates maximum efficacy that is postulated as greater than that possible using standard second generation approaches. We used a combined method of optimizing TCE mediated synaptic distance and apparent affinity tuning of the TAA binding arms to generate a relatively long but persistent synapse that supports a wide cytokine window, potent killing and a reduced propensity towards immune exhaustion. Importantly, this next generation TCE induced significant tumor growth inhibition *in vivo* but unlike a first-generation non-decoupled benchmark TCE that induced lethal CRS, no signs of adverse events were observed.

## Introduction

Tumor treatment using T cell engagers (TCE)s, a novel class of multi-specific therapeutic proteins has emerged as a promising strategy in the field of cancer immunotherapy (1). These biologics are designed to redirect the specificity and activate the patient’s own T cells to selectively recognize and eliminate tumor cells that express a targeted tumor associated antigen (TAA). Simultaneous binding of a cytotoxic T lymphocyte to a cancer cell creates a cytolytic bridge that resembles an immunological synapse (termed a “lytic synapse”) (2). The attached cancer cells can then be killed via perforin and granzymes released by T cells (3–6) or bystander mechanisms such as Fas-FasL interactions that are dependent on the release of IFNγ (6). TCEs have demonstrated clinical proof of concept for hematological malignancies and hold promise for solid tumors. As such, only TCEs targeting hematologic malignancies have been granted FDA approval. These consist of one CD19 targeted dual-scFv CD3 Bispecific T cell engager (BiTE), blinatumomab, for the treatment of lymphoblastic leukemia (7–9), two CD3 bispecific antibodies (bsAb) targeting BCMA, teclistamab and elranatamab-bcmm, for the treatment of relapsed or refractory multiple myeloma (10, 11), three TCEs targeting CD20, mosunetuzumab, glofitamab, and epcoritamab (12–14) and the GPRC5D targeted TCE talquetamab (15). Toxicity remains a challenge as this class of TCEs commonly induce cytokine release syndrome (CRS) (16) and concomitant neurotoxicity. In addition to these adverse events for which progress has been made to reduce the severity, potentially devastating on-target/off-tumor toxicity remains perhaps the biggest obstacle should the TAA be expressed on a vital organ (17, 18), common in the solid tumor setting. This is especially concerning given that higher peripheral concentrations of TCEs may be required to penetrate solid tumors to induce sufficient exposure to achieve an efficacious dose (1, 19). These common adverse events and dose limiting toxicities highlight the importance of careful monitoring and management strategies and an unmet need for next generation TCEs.

Engineering TCEs that “decouple cytotoxicity from cytokine release” is considered a high value mitigation strategy. But, to date, such TCEs generally lack a “cytokine window” consisting of a dose range in which maximal killing is reached but cytokine release can be modulated from very little for safety to levels sufficient to mediate “bystander killing”. Instead, the vast majority of these second generation TCEs induce low cytokine release at the dose needed to achieve maximal killing and little is known about the propensity of these TCEs to induce immune exhaustion, which is likely more critical in the solid tumor setting in which T cell infiltration of the tumor is limited. Moreover, bystander killing may be critical to achieving efficacy beyond partial response due to the high degree of heterogeneity of targeted TAA expression including tumor cells altogether devoid of the TAA. These outstanding questions may account for the current controversy as to whether TCEs can demonstrate efficacy in the solid tumor landscape.

The major strategies to “decouple cytotoxicity from cytokine release” have been centered around (a) detuning TCEs via lowered apparent affinity for CD3 (20–22), (b) targeting a “unique” epitope on CD3 (20, 23), (c) using low affinity/avidity based binding to the TAA to “mimic T cell Receptor like binding” (22, 24) and (d) simultaneous affinity modulation of both targets (25).

Another method, which is a basis for this study, is modulation of the geometry of the TCE, which has been shown to alter TCE potency by impacting of T cell/tumor cell intermembrane characteristics. These include the magnitude of immune-regulatory proteins in the synapse (e.g. CD45 and CD148), which is hypothesized to result from alterations in the intermembrane distance and the strength of the synapse formed between the T cell and tumor cell (26–30). The paradigm that (a) the strength of an immunological synapse (31) and (b) the spatial organization of activating, adhesion and inhibitory receptors within the synapse (i.e. the “kinetic-segregation model”) dictate the temporal cytotoxic and pro-inflammatory phenotype of T cells is well described (32–34). However, only a few studies have provided evidence that manipulation of TCE geometry/bridge size or targeting epitopes at different distances from the tumor cell membrane, impact the magnitude of cytotoxicity and cytokine release (26, 29, 30, 35). For example, Bluemel et al. and Chen et al. observed that the cytotoxic potency of TCEs was enhanced when engineering the TCE targeted TAA epitope from EpCAM or BCMA, respectively, closer to the tumor plasma membrane (26, 35). Unfortunately, none of these reports directly investigated the impact of synaptic bridge distance and TCE geometry on the generation of a cytokine window. Further, since intermembrane or synaptic distances have not been directly measured by others, are beyond the scope of this work, and context dependent components of the synapse (e.g. adhesion molecules) can also impact TCE function, we will term this parameter “apparent synaptic range” for the purposes of this study.

Here we describe the generation of bispecific CD3 x ROR1 TCEs in a 2 + 1 format for which protein geometry, TAA epitope selection and bridge strength are all modulated to generate a TCE with a large cytokine window. All TCEs were built on an IgG Fc knob-into-holes scaffold (36) to extend half-life and were rendered effector null via incorporation of the LALA mutation (37, 38).

ROR1, a receptor tyrosine kinase, was chosen as the proof-of-concept TAA because (a) its domain structure is well described (39), (b) antibodies specific for the membrane proximal Kringle domain (clone R11), the intermediate distanced Frizzled domain (clone R12) (40), and the distal Ig domain (2A2) (41) are readily available, and (c) TCEs incorporating R12 or R11, which have been described to demonstrate distinct potencies that align with the “kinetic-segregation model”, can be used as benchmarks (40, 42, 43). ROR1 is expressed on a large array of both solid and liquid tumor cells (44), and thus represents a high value target. ROR1’s expression pattern on normal tissues is controversial and may include some organs such as pancreas, gut and liver (44). This makes the TAA particularly suitable as a case study for decoupling of cytotoxicity from cytokine release in a manner that results in a large cytokine window.

## Materials and methods

### Human peripheral blood mononuclear cell isolation

PBMCs were isolated from whole blood from healthy donors using Ficoll-Paque Plus medium. In brief, 35 mL of diluted whole blood (1 volume of whole vs 1 volume of PBS) was gently overlayed on the top of 15 mL Ficoll-Paque Plus medium without disturbing the interface in a 50-mL conical tube. After centrifuging for 40 min at 400 × g at room temperature without brake, the buffy coat (interface layer between Ficoll and serum) was collected and diluted in 5 volumes of PBS. After centrifuging for 5 min at 500 × g at room temperature, PBMCs were resuspended in PBS and washed once in PBS by centrifuging for 5 min at 500 × g at room temperature. PBMCs were then resuspended in 5 mL of ACK lysis buffer and incubated for 5 minutes at room temperature to remove red blood cell residues. After 5-minute’s incubation, 45 mL PBS was added to PBMCs and centrifuged for 15 min at 100 × g at room temperature. At last, PBMCs were resuspended in culture medium (RPMI1640 with 10% FBS and 1% penicillin/streptomycin) for cytotoxicity and IFN gamma release assay setting up.

### Human T cell isolation, activation, expansion, and recovery

PBMCs were isolated from whole blood from healthy donors following the procedure described above. T cells were isolated from fresh human PBMCs using EASAYSEP human T cell isolation kit from STEMCELL. In Brief, PBMCs were mixed with isolation antibody cocktail (provided with the kit) in Ca2+- and Mg2+-free PBS containing 2% FBS and 1 mM EDTA. After 5-minute incubation at room temperature, RapidSpheres™ bead (provided with the kit) was added to the mixture and then immediately assembled on EASY50 EASY SEP magnet from Stemcell. After 10-minute incubation at room temperature, cells in mixture solution were collected and assembled on EASY50 EASY SEP magnet from Stemcell again. After 5-minute incubation at room temperature, purified T cells in mixture solution were collected and pelleted by centrifugation. Purified T cells were frozen down and kept in liquid nitrogen for further usage.

To activate purified T cells, isolated T cells were resuspended in ImmunoCult™-XF T cell expansion medium from Stemcell. ImmunoCult™ human CD3/CD28 T cell activator from Stemcell and 100 U/mL human IL-2 from R&D System were added to the T cells to activate for 3 days for activation and 12 days for expansion. During the 12-days expansion, fresh ImmunoCult™-XF T cell expansion medium with 100 U/mL human IL-2 was added to the cells whenever the cell density was above 1 million cells per milliliter. At the end of 12-day expansion, expanded T cells were frozen down and kept in liquid nitrogen.

To recover T cells, frozen activated T cells or T cells without activation were recovered from liquid nitrogen and thawed in 37°C water bath. The recovered T cells were resuspended in RMPI1640 medium with 10% heat-inactivated FBS with 250 U/mL human IL-2. After 48-hour incubation at 37°C, T cells were ready for further usage.

### Human CD8+ T cell isolation, activation, and recovery

PBMCs were isolated from whole blood from healthy donors following the procedure described above. CD8+ T cells were isolated from fresh human PBMCs using EASAYSEP human CD8+ T cell isolation kit from STEMCELL. In Brief, PBMCs were mixed with isolation antibody cocktail (provided with the kit) in Ca2+- and Mg2+-free PBS containing 2% FBS and 1 mM EDTA. After 5-minute incubation at room temperature, RapidSpheres™ bead (provided with the kit) was added to the mixture and then immediately assembled on EASY50 EASY SEP magnet from Stemcell. After 10-minute incubation at room temperature, cells in mixture solution were collected and assembled on EASY50 EASY SEP magnet from Stemcell again. After 5-minute incubation at room temperature, purified CD8+ T cells in mixture solution were collected and pelleted by centrifugation. Purified CD8+ T cells were frozen down and kept in liquid nitrogen for further usage.

To expand CD8+ T cell, purified human CD8+ T cells were resuspended in ImmunoCult™-XF T cell expansion medium from Stemcell. ImmunoCult™ human CD3/CD28 T cell activator from Stemcell and 100 U/mL human IL-2 from R&D System were added to the T cells to activate for 12 days. During the 12-days expanded, fresh ImmunoCult™-XF T cell expansion medium with 100 U/mL human IL-2 was added to the cells whenever the cell density was above 1 million cells per milliliter. At the end of 12-day expansion, expanded CD8+ T cells were frozen down and kept in liquid nitrogen.

To recover CD8+ T cells, expanded CD8+ T cells or CD8+ T cells were recovered from liquid nitrogen and thawed in 37°C water bath. CD8+ T cells were resuspended in RMPI1640 medium with 10% heat-inactivated FBS with 250 U/mL human IL-2. After 48-hour incubation at 37°C, CD8+ T cells were ready for further usage.

### Cytotoxicity assay

On the day before assay setting up, selective antibiotics were removed from target cell lines. On the day of assay setting up, target cell lines were collected by brief TrypLE treatment and then washed with culture medium by centrifuge at 500 ×g for 5 min at room temperature. Target cells were then resuspended in culture medium to determine the viability by trypan blue exclusion on Cellometer. The viable cell density was adjusted to 50,000 cells/mL in culture media. 100 uL target cell suspension (5000 target cell) was carefully dispensed to each well of a 96-well black clear flat-bottom tissue culture plate using multichannel pipettor. The plate was then incubated for 4-5 hours in tissue culture incubator to make sure that the target cells were fully attached to the bottom of the 96-well plate.

Fresh PBMCs or recovered T cells were pelleted down by centrifuge for 5 min at 500 × g at room temperature and resuspended in culture medium. The viability of cells was also determined via trypan blue exclusion. The viable PBMC density was adjusted to 3 million cells/mL in RPMI1640 medium with 10% FBS and 1% penicillin/streptomycin while the viable T cell density was adjusted to 0.5 million cells/mL in RPMI1640 medium with 10% FBS and 1% penicillin/streptomycin. After 4-5 hours’ incubation, culture medium was carefully removed from the 96-well plates with target cells. 50 uL of 3 million cells/mL PBMCs suspension (150,000 PBMCs) or 50 uL of 0.5 million cells/mL PBMCs suspension (25,000 T cells) was added to the designated well in the 96-well plate with target cell, which would result in the E:T ratio of 30:1 for PBMC and 5:1 for T cells. For other E:T ratio, the effector cells were resuspended in RPMI1640 medium with 10% FBS and 1% penicillin/streptomycin at certain density to yield needed effector cell number in 50 uL cell suspension.

CD3xROR1 T-cell-engagers (TCEs) were prepared and serially diluted (5-fold serial dilution) in RPMI1640 medium with 10% FBS and 1% penicillin/streptomycin ranging from 200 nM to 2.56 pM. 50 uL of prepared CD3xROR1 TCEs at different concentrations was then added to the designated wells in the 96-well plate with PBMC and target cells and incubated for one, two or three days at 37°C with 5% CO2. At the end of incubation, the 96-well plates were centrifuged for 1 minute at 500 ×g to transfer 50μL of supernatant to V-bottom storage plate using a multichannel pipettor for IFN gamma release assay. ONE-Glo Luciferase Assay solution was brought to room temperature. 50 uL of One-Glo solution was then added to the designated well and incubated for 2 min at room temperature. The bioluminescence was measured on a plate reader with preset Bio-luminance protocol. The killing percentage was calculated using the formula: killing percentage = 100* (bioluminescence intensity of target cell alone - bioluminescence intensity of sample)/(bioluminescence intensity of target cell alone - bioluminescence intensity of PBMC alone).

### IFN gamma release detection

IFN gamma release from cytotoxicity assay was measured using human IFN gamma ELISA detection kit. In brief, coating antibody provided with the kit was diluted to suggested concentration by following the protocol provided by the kit manufacturer. 100 ul of diluted coating antibody was added to the Nunc MaxiSorp flat-bottom 96-well plate, sealed plate and incubated at 4°C overnight. The plates were then washed 4 times with Wash Buffer. To block non-specific binding and reduce background, 200 μL Assay Diluent A was added and incubated the plate at room temperature for 1 on a plate shaker (400 rpm). After blocking, the plates were washed 4 times with Wash Buffer. 100 μL/well of standards (prepared with culture medium) or samples were then added to the designated wells and incubated at room temperature for 2 hours on a plate shaker (400 rpm). After 2-hour incubation with samples or standards, the plates were washed 4 times with Wash Buffer and 100 μL of diluted Detection Antibody solution was added to incubate at room temperature for 1 hour on a plate shaker (400 rpm). The plates were then washed 4 times with Wash Buffer and 100 μL of diluted Avidin-HRP solution was added to incubate at room temperature for 30 minutes on a plate shaker (400 rpm). After 30-minute incubation with Avidin-HRP, the plates were washed 5 times with Wash Buffer and 100 μL of freshly mixed TMB Substrate Solution was added to incubate at room temperature for 20 minutes in the dark. 100 μL of Stop Solution was then added to the well to stop the reaction. The absorbance at 450 nm was measured on a plate reader and the concentration of IFN gamma in each sample was calculated by using the standard curve generated with the absorbance of different concentration of standards.

### Cell binding assay

2*10^5^ target cells were seeded in each well in a V-shaped 96-well plate and twice washed with 200 µL PBS by centrifugation at 500 xg for 5 minutes at room temperature. Afterward, the cells were incubated in 100 µL of Live/Dead fixable green labeling buffer (ThermoFisher Scientific) for 15 minutes in the dark. After washing twice with 200 µL PBS, the cells were incubated in 50 µL BD staining buffer (BD Biosciences) with 10% goat serum at room temperature for 20 minutes. Afterwards, 50 µL BD staining buffer with the appropriate amount of T cell engager was added. After incubation for 60 min at 4°C in the dark, the cells were washed twice with 200 µL PBS. Then the cells were stained in 100 µL BD staining buffer with goat anti-human IgG-Alexa Fluor 647 antibody (final concentration: 2.5 µg/mL). After washing twice with 200 µL PBS, cells were analyzed on iQUE Screener. Data were analyzed with FlowJo and the gating strategy was followed: In brief, target cell population was gated by FSC and SSC. Single cells were selected from target cell population by FSC-A and FSA-H. Living single cells were gated from single cells by live/dead green dye low. MFI of expressed target was acquired in FlowJo. Binding EC50 was generated using MFI from FlowJo and analyzed in GraphPad Prism using one site-specific binding non-linear logistic regression.

### PD-1, TIGIT, and CD69 detection on T cells

Following the experiment setting up for cytotoxicity assay, target cells were added to the wells (5,000 target cells per well) in 96-wells plate and incubated for 5 hours. After 5-hour incubation, cell culture medium was removed from the plate without touching the target cells and 50 uL of fresh PBMCs (150,000 PBMCs per well) were added to the wells with target cells (E:T ratio 0f 30:1). 50 uL of CD3xROR1 TCEs were then added to the designated wells with 150,000 PBMCs and 5,000 target cells. After incubating at 37°C for 24, 48 or 72 hours, PBMCs were collected and washed once with PBS by centrifugation at 500 ×g for 5 minutes at room temperature and added to V-bottom 96-well plate. To prepare Live/Dead fixable red, one vial of powder was dissolved into 50 uL of DMSO to make stock solution and then diluted 1 uL of stock Live/Dead fixable red in 1 mL of PBS to prepare the working solution. 100 uL of Live/Dead fixable red working buffer was then added to the cell in the V-bottom 96-well plate and incubated for 15 minutes at room temperature. After the 15-minute incubation, the cells were washed twice with PBS by centrifugation at 500 g for 5 minutes at room temperature. The cells were then cultured with antibodies (PE-Cy5-conjugated mouse anti-human TCRa/b antibody, PE-conjugated mouse anti-human CD19 antibody, BB515-conjugated mouse anti-human CD56 antibody, BB700-conjugated mouse anti-human CD14 antibody, PE-Cy7-conjugated mouse anti-human PD-1, TIGIT or CD69 antibody) in 100 uL of Biolegend staining buffer at 4°C for 45 minutes. After the 45-minute incubation with antibody, the cells were washed with PBS by centrifugation at 500 g for 5 minutes at room temperature and resuspended in 100 uL of BioLegend fixation buffer. The samples were then analyzed on Cytek flow cytometer. Data were analyzed with FlowJo and the gating strategy was followed: In brief, target cell population was gated by FSC and SSC. Single cells were selected from target cell population by FSC-A and FSA-H. Living single cells were gated from single cells by live/dead red dye low. T cells were gated from living single cells by TCRa/b high. PD-1, TIGIT and CD69 expression was then gated on T cells with PD-1, TIGIT or CD69 high.

### Bystander killing assay

On the day before assay setting up, selective antibiotics were removed from target cell lines (ROR1+ MDA-MB-231 RFP cell or ROR1- T-47D eGFP FLUC cell). On the day of assay setting up, target cell lines were collected by brief TrypLE treatment and then washed with culture medium by centrifuge at 500 ×g for 5 min at room temperature. Target cell lines were then resuspended in culture medium to determine the viability by trypan blue exclusion on Cellometer. The viable cell density was adjusted to 50,000 cells/mL in culture media. 100 uL ROR1- target cell suspension (5000 cell) was carefully dispensed to the designated well of a 96-well black clear flat-bottom tissue culture plate using multichannel pipettor for the group with ROR1- target cell only. 100 uL ROR1+ target cell suspension (5000 cell) was dispensed to the designated well of the 96-well black clear flat-bottom tissue culture plate for the group with ROR1+ target cell only. For the group with both ROR1+ and ROR1- target cells, 100 uL ROR1- target cell suspension (5000 cell) and 100 uL ROR1+ target cell suspension (5000 cell) was added to the designated well of the 96-well black clear flat-bottom tissue culture plate. The plate was then incubated for 4-5 hours in tissue culture incubator to make sure that the target cells have attached to the bottom of the 96-well plate.

Recovered T cells were pelleted down by centrifuge for 5 min at 500 × g at room temperature and resuspended in culture medium. The viable T cell density was adjusted to 0.5 million cells/mL in RPMI1640 medium with 10% FBS and 1% penicillin/streptomycin. After 4-5 hours’ incubation, culture medium was carefully removed from 96-well plates with target cells. 50 uL of 0.5 million cells/mL T cell suspension (25,000 T cells) was added to the designated well in the 96-well plate with target cell, which would result in the E:T ratio of 5:1 for T cells.

CD3xROR1 T-cell-engagers (TCEs) were prepared and serially diluted (5-fold serial dilution or 10-fold serial dilution) in RPMI1640 medium with 10% FBS and 1% penicillin/streptomycin ranging from 200 nM to 2.56 pM. 50 uL of prepared CD3xROR1 TCEs at different concentrations was then added to the designated wells in the 96-well plate with T cells and target cells and incubated in CELLINK CELLCYTE X at 37°C with 5% CO2. Cell number of ROR1+ MD-MB-231 RFP cell and ROR1- T-47D eGFP FLUC cell in each well was monitored every three hours for 72 hours. The change of target cell number in each well was plotted against time and the killing percentage of target cell was calculated by using target cell number at the time zero as the 100% base.

### Serial killing assay

On the day before assay setting up, selective antibiotics were removed from target cells. On the day of assay setting up, target cell lines were collected by brief TrypLE treatment and then washed with culture medium by centrifuge at 500 ×g for 5 min at room temperature. Target cell lines were then resuspended in culture medium to determine the viability by trypan blue exclusion on Cellometer. The viable cell density was adjusted to 200,000 cells/mL in culture media. A serial dilution of target cell (factor 2) was made from 200,000 cells/mL to 3,125 cells/mL (200,000 cells/mL, 100,000 cells/mL, 50,000 cells/mL, 25,000 cells/mL, 12,500 cells/mL, 6,250 cells/mL, 3,125 cells/mL, 0 cell/mL) with culture medium. 50 uL target cell suspension at different densities was carefully dispensed to designated standard control wells of a 96-well black clear flat-bottom tissue culture plate using multichannel pipettor, which yielded 10,000 cells, 5,000 cells, 2,500 cells, 1,250 cells, 625 cells, 313 cells, 156 cells, 0 cell per well as standard controls. For all testing article wells, 100 uL target cell suspension at the density of 50,000 cell/mL (5,000 cell per well) was carefully dispensed to designated wells. The plate with target cells were incubated at 37°C for 4 hours.

After 48-hour recovery in 250 U/mL IL-2, activated CD8+ T cells were pelleted down by centrifuge for 5 min at 500 × g at room temperature and resuspended in culture medium. The viability of cells was also determined via trypan blue exclusion. The viable recovered CD8+ T cells were adjusted to 800,000 cells/mL in culture medium. A serial dilution of activated CD8+ T cell (factor 2) was made from 800,000 cells/mL to 6,250 cells/mL (800,000 cells/mL, 400,000 cells/mL, 200,000 cells/mL, 100,000 cells/mL, 50,000 cells/mL, 25,000 cells/mL, 12,500 cells/mL, 6,250 cells/mL) with culture medium. 50 uL recovered activated CD8+ T cell suspension at different densities was carefully dispensed to designated wells with 5,000 target cells for testing articles or controls in a 96-well black clear flat-bottom tissue culture plate using multichannel pipettor, which yielded 40,000 cells (E:T ratio of 8:1), 20,000 cells (E:T ratio of 4:1), 10,000 cells (E:T ratio of 2:1), 5,000 cells (E:T ratio of 1:1), 2,500 cells (E:T ratio of 1:2), 1,250 cells (E:T ratio of 1:4), 625 cells (E:T ratio of 1:8), 313 cells (E:T ratio of 1:16) per well for all testing articles or controls.

Testing articles and proper negative controls were prepared in culture medium at the concentration of 1 nM. 100 uL of prepared testing articles of negative controls was added to the designated wells with 5,000 target cells and different number of activated CD8+ T cells. For the wells with different number of target cells as standard controls, 150 uL of culture medium was added to bring the total volume to 200 uL. The cells were incubated for one day at 37°C with 5% CO2. At the end of incubation, the 96-well plates were centrifuged for 1 minute at 500 ×g to transfer 150μL of supernatant to V-bottom storage plate using a multichannel pipettor for further usage. ONE-Glo Luciferase Assay solution was brought to room temperature. 50 uL of One-Glo solution was then added to the designated well and incubated for 2 min at room temperature. The bioluminescence was measured on a plate reader with preset Bio-luminance protocol. To calculate the killing frequency, the bioluminescence intensity of target cell at different seeding numbers as standard controls were plotted against the cell numbers to generate a standard curve. The number of living target cells in each testing sample wells were calculated with the standard curve by using the bioluminescence intensity of each testing sample well. The killed target cell number was calculated by subtracting the calculated living target cell number for each testing sample from 5000 (plating number of target cells). The killing frequency of target cells in each well was generated by dividing killed target cell number with the input CD8+ T cell number for the same well.

### Mouse xenograft tumor model in humanized immunocompromised mice

The capacity of the ROR1 x CD3 bsAbs to mediate *in-vivo* tumor growth inhibition (TGI) was assessed using the human ROR1+ xenograft TNBC mouse tumor model, MDA-MB-231. Briefly, NSG (NOD-scid IL2Rgammanull; Jackson Laboratory) mice were injected subcutaneously with 5 million MDA-MB-231 admixed in Matrigel Matrix (Corning) into the right dorsal flanks of the animals. On the same day, the mice were also injected intravenously with 5 million expanded human T cells. Expanded T cells for injection were selected from several healthy donors based on minimal alloreactivity towards MDA-MB-231. In short, T cells from two healthy donors were expanded using ImmunoCult™ human CD3/CD28 T cell activator reagent (Stemcell) as per the manufacturer’s recommended protocol. Alloreactivity was examined using our *in-vitro* bioluminescent cytotoxicity and IFNγ release assays (as described above) and was defined as maximum killing equal to 15% or less and maximum IFNγ release equal to 300 pg/ml or less at an E:T of 5:1. When tumor volumes reached between 75-100 cubic millimeters, the mice were randomized into 5 groups. Group 1, consisting of 5 mice, received mock treatment (PBS) twice weekly. Groups 2-4 consisted of 10 mice each and received two weekly doses of hum-VHH-2-LC at 0.03 mg/kg, 0.3 mg/kg or 3 mg/kg. Group 5 consisted of 7 mice and received two weekly doses of R11-scFv-1 at 3 mg/kg. Tumor volumes were measured every 3-5 days and the survival of mice was also recorded. Mice were sacrificed at the end of the study.

### Decoupling ratio calculation

Cytotoxicity and IFN gamma release were plotted against the concentration of TCE in GraphPad Prism. For this purpose, the following equation was used:


$$y=Bottom+x^{Hillslope}\;\frac{Top-Bottom}{x^{Hillslope}+EC50^{Hillslope}}$$

Area under the curve (AUC) for cytotoxicity and IFN gamma release was calculated using the AUC algorithm in Prism. The decoupling ratio was generated by the following equation:


$$decoupling\;ratio\;=\;\frac{AUC_{killing}\times \text{log}(EC50_{IFN})\;}{AUC_{IFN}\times \text{log}(EC50_{killing})}$$

**Figure 1:**
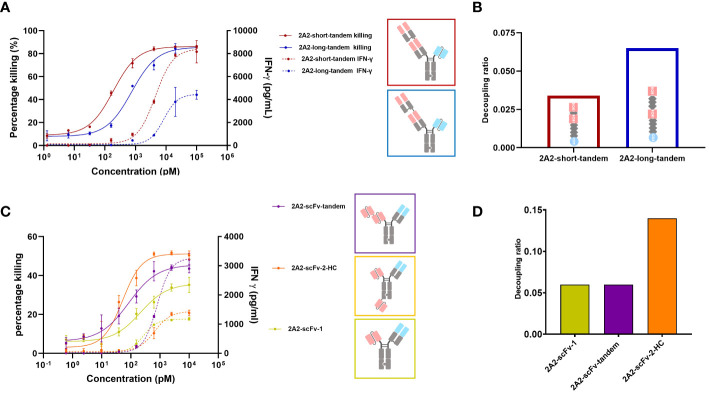
Decoupling of tumor cell killing from IFNγ release induced by different 2A2 harboring T cell engager constructs is dependent on the geometrical arrangement of the binding moieties. The capacity of two CD3 x ROR1 bispecific antibodies (bsAbs) to induce T cell-mediated killing of tumor cells (solid line) and IFNγ release (dashed line) were measured by mixing PBMCs with ROR1+ MDA-MB-231 cells at the E:T ratio of 30:1. 48 hours after the addition of TCEs, the killing of tumor cells and IFNγ release was measured in duplicates. All assays were repeated in at least two donors and representative results are shown. Error bars are based on the SD. **(A)** The two constructs (2A2-short-tandem and 2A2-long-tandem) contained two 2A2 Fab fragments targeting the Ig domain of ROR1 and one CD3 binding scFv (IF3-3). The linker length between the ROR1 binding moieties was 10 amino acids (aa) for 2A2-short-tandem (red) and 20 aa for 2A2-long-tandem (blue). **(B)** Decoupling ratio of the two constructs. The decoupling ratio was defined as the ratio of the AUC of the target cell killing multiplied by the log of the EC50 for IFNγ release divided by the AUC of the IFNγ release multiplied with the log of the EC50 for cell killing. **(C)** Three different bispecific constructs containing 2A2 scFv and CD3 binding Fab were also tested for tumor cell killing and IFNγ release. The construct 2A2-scFv-tandem (purple) with two ROR1 binding scFv moieties linked together induced the greatest amount of IFNγ release. The construct 2A2-scFv-2-HC (orange) with one scFv fused to the C-terminus of the heavy chain showed the most potent killing of target tumor cells. The construct consisting of only one scFv (yellow) showed the lowest killing potency. **(D)** 2A2-scFv-1 and 2A2-scFv-tandem both had a decoupling ratio of about 0.06. The decoupling ratio of 2A2-scFv-2-HC was approximately 0.14.

Except for **Figure 1**, decoupling ratios were normalized to certain non-decoupling testing articles to compare the variation among different experiments. Experimental data for calculation of decoupling ratios are provided in the supplementary Excel sheet.

## Results

### Design of different ROR1 x CD3 formats

It was previously reported that TCE mediated apparent synaptic range impacts both cytotoxicity and cytokine release such that a shorter distance between T cell and tumor cell appears to be associated with stronger T cell activation (26, 29, 30, 35). Chen et al. found that cytotoxicity mediated by a BCMA targeting TCE decreased from ~80% to ~0% as the distance between tumor cell membrane and BCMA ectodomain increased (26). Li et al. observed no tumor cell killing with a CD3 x FcRH5 bsAb targeting a distal epitope (gD), unless FcRH5 was truncated, leading to strong cytotoxicity (29). This suggests that T cell cytotoxicity depends on the intermembrane distance, with shorter distances enhancing T cell activity. Thus, we first investigated the impact of extending the apparent synaptic range on decoupling cytotoxicity from cytokine release. We were particularly interested in assessing whether one could convert a TCE that does not or poorly decouple cytotoxicity from cytokine release into one that does. The ROR1 antibody 2A2 (KD = 32.6 nM) described by Baskar et al. (41) targeting the membrane distal Ig domain of human ROR1 was chosen for this purpose and several TCE formats were examined. Two 2A2 Fab regions were assembled on an ADCC-silenced Fc including inter-Fab linkers of different lengths and rigidity. For the construct termed “short-2A2-tandem”, a (G4S)_2_ linker was utilized and for the construct termed “long-2A2-tandem”, a rigid (EAAAK-G4S)_2_ linker was used. The CD3 binding moiety, 1F3-3 (45), was placed as a single-chain variable fragment (scFv) on the other arm for T cell engagement. The generation of heterodimers was facilitated via knobs into holes technology. As shown in **Figure 1A**, the geometric arrangement of the ROR1 binding moieties impacts the release of IFNγ from T cells and their capacity to kill tumor cells in a ROR1 dependent fashion. 2A2-short-tandem induced T cell-mediated killing of ROR1-positive MDA-MB-231 tumor cells with a potency (EC50) of 189 pM and efficacy (Emax) of 86.2%. 2A2-long tandem showed a moderately reduced potency of 751 pM but maintained efficacy (85.6%). The difference, however, was appreciable for IFNγ release. 2A2-long-tandem induced a significantly lower release of IFNγ with a EC50 of 8.4 nM and an IFNγ release Emax of 4,400 pg/mL whereas the EC50 and Emax for 2A2-short-tandem was 4.5 nM and 8,400 pg/mL, respectively. In this case, consistent with multiple reports (24, 25, 46), decoupling of cytotoxicity from cytokine release was mainly restricted to the maximum release of IFNγ. 2A2-long-tandem showed a higher decoupling ratio of 0.065 in comparison to 2A2-short tandem (0.034) (**Figure 1B**). Next, three other TCEs were generated. The ROR1 binding module of these TCEs consisted of the variable light chain (VL) and variable heavy chain (VH) domains of 2A2 linked with a (Gly4Ser)3 linker in a scFv version. This enables further formats for tandem arrangement and, as for 2A2-scFv-2-HC, placement of one ROR1 binding module on the N-terminus and the other one to the C-terminus of Fc scaffold. As above, the T cell binding module was the CD3 specific 1F3-3 Fab in all constructs (**Figure 1C**). The two trivalent TCEs consisting of two ROR1 binding scFvs showed enhanced target tumor cell killing in comparison to 2A2-scFv-1, with 2A2-scFv-2-HC having the highest killing Emax. This result highlights and supports the positive influence of avidity of ROR1 binding moieties on potency (41). 2A2-scFv-tandem showed the highest release of IFNγ. The other trivalent TCE, 2A2-scFv-2-HC, in contrast showed similar IFNγ release to the bivalent 2A2-scFv-1. 2A2-scFv-2-HC displayed the highest decoupling ratio of 0.14 (**Figure 1D**).

**Figure 2:**
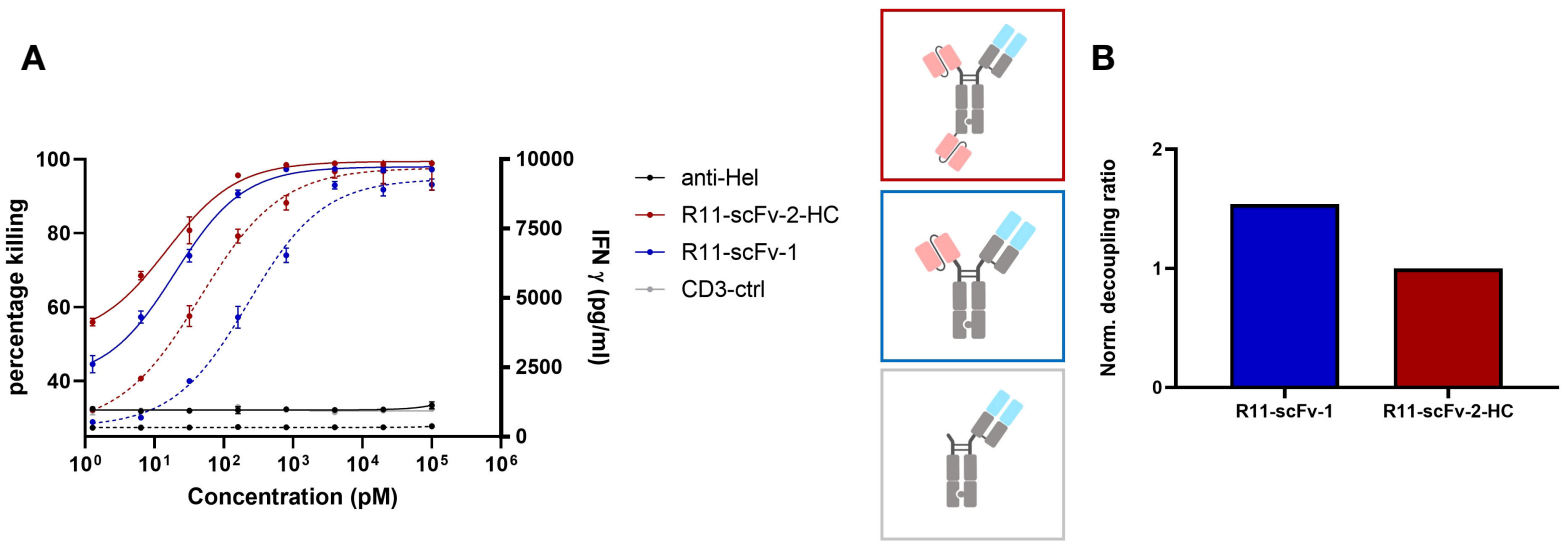
A T cell engager binding closer to the membrane could not decouple tumor cell killing from IFNγ release. Two constructs harboring the scFv derived from ROR1 binder R11, which binds ROR1 close to the cell membrane in the Kringle domain, were also tested for tumor target cell killing and IFNγ release. Expanded T cells purified from healthy donors were used as effector cells at an E:T ratio of 3:1. Both assays were read after 48 hours, in duplicates. Error bars are based on the SD. A monospecific construct control containing only 1F3 3 CD3 Fab without ROR1 binding moiety, and an anti-HEL antibody were used as negative controls. **(A)** The construct with one and two R11 moieties showed comparable efficacy (Emax) pertaining to tumor cell killing and IFNγ release. R11-scFv-1 had a slightly lower potency (EC50) for killing and IFNγ release. **(B)** The trivalent R11-scFv-2-HC showed comparable decoupling capacity to R11-scFv-1, which consisted of only one ROR1 binding moiety. The decoupling ratios were normalized to R11-scFv-2-HC. All assays were repeated in at least two donors and representative results are shown.

Next, we used the anti ROR1 antibody clone R11 for the construction of T cell engagers (40). R11 recognizes an epitope close to the cell membrane (kringle domain). A TCE reported by Qi and colleagues incorporating this clone was observed as highly potent and efficacious in mouse tumor models (36) and therefore served as the first generation TCE benchmark. We designed a construct (R11-scFv-1) with one ROR1 binding moiety (similar to that generated by Qi et al.) or two ROR1 binding motifs (R11-scFv-2-HC). In the R11-scFv-2-HC construct, similar to 2A2-scFv-2-HC, the second single-chain variable fragment was fused to the C-terminus of the heavy chain. These two constructs were tested for T cell-mediated killing of tumor cells and release of IFNγ (**Figure 2A**). R11-2-scFv displayed a slightly higher potency for T cell-mediated cytotoxicity, compared to its single ROR1 binding counterpart but the same killing Emax. Both constructs were associated with high amounts of released IFNγ, even at low concentrations and displayed a normalized decoupling ratio below 2. Thus, our first generation benchmark TCE also served as the non-decoupled control (**Figure 2B**). Given that TCE 2A2-scFv-2-HC with the same format (described above) demonstrated characteristics of a second generation decoupled TCE, these data sets indicate that not only the spatial arrangement of binding sites relative to each other, but also epitope localization relative to the cell membrane play crucial roles in decoupling of cytotoxicity from cytokine release.

### Design of VHH-based TCEs

Since the TCEs designed above using the anti-ROR1 clone 2A2 showed decoupling capacity but low potency and a minimal cytokine window, we searched for new ROR1 binders. VHHs, single domain antibodies from camelids, also termed nanobodies, are well described as being capable of recognition of a larger array of epitopes versus traditional antibodies (47). Such VHHs are highly stable and can be used in a plug and play manner for the construction of diverse formats (48). Thus, we focused on the generation of ROR1 specific TCEs with optimal decoupled properties using nanobodies. To obtain VHH binders that bind the distal region of ROR1 with the hypothesized optimal distance from the cell membrane, (based on our results with clone 2A2 above), a Bactrian camel was immunized first with full length ROR1 protein and then boosted with protein consisting only of the Ig-like domain of ROR1. Candidate VHHs were identified from a phage display library. After screening a large array of VHH (data not shown), we identified clone 5A1, which bound to human ROR1 inside the Ig-like domain with an apparent affinity of ~20 nM (**Supplementary Figure S1**) for further investigation. Based on this VHH, four different T cell engagers consisting of two ROR1 targeting VHHs one CD3 binding Fab were designed. The positions of (a) the CD3 binder, IF3-3, which has a relatively high apparent affinity of ~10 nM for CD3ϵ (**Supplementary Figure S2**), and (b) of one of the two 5A1 VHHs on the N-terminal of the hinge directly opposite the CD3 binding fab, were kept constant to avoid the introduction of multiple variables for engineering a TCE with a wide cytokine window. Thus, the constructs differed only in the position of the second VHH binding module.

**Figure 3:**
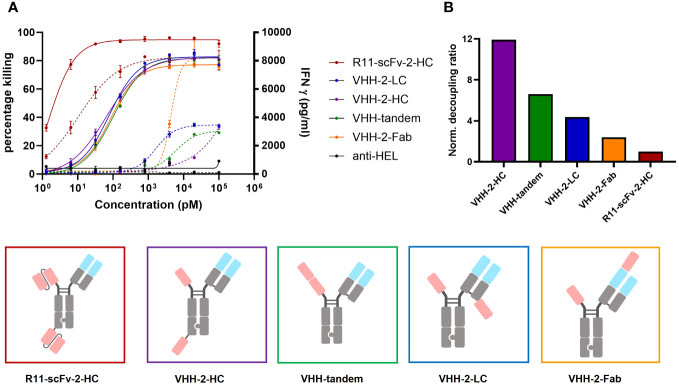
Comparison of tumor cell killing and IFNγ release of different T cell engagers containing the ROR1 binding VHH 5A1. Measurements were made in duplicate and error bars are based on the SD. **(A)** Four T cell engagers in which the position of their second 5A1 VHH was varied showed comparable target tumor killing but the amount of released IFNγ varied depending on the geometrical arrangement of the second 5A1 VHH. VHH-2-Fab, where the 5A1 VHH was fused to the CD3 binding Fab region showed the highest level of IFNγ release. VHH-2-HC showed the lowest amount of released IFNγ. In the assays, PBMCs were used as effector cells at an E:T ratio of 30:1 and both assays were read at 48 hours. **(B)** The decoupling ratio was calculated as described above and normalized to the non-decoupled R11-scFv-2-HC. VHH-2-HC And VHH-2-Fab had the highest and lowest decoupling ratios, respectively. All assays were repeated in at least two donors and representative results are shown.

All four constructs showed comparable T cell-mediated killing of ROR1+ tumor cells (**Figure 3A**). In comparison to the 2A2 based constructs, they all display increased T cell mediated cytotoxicity with Emax values of about 80%. However, we observed appreciable differences among the constructs regarding IFNγ release. VHH-2-Fab shows the highest level of IFNγ release, followed by VHH-2-LC and VHH-2-tandem. VHH-2-Hc showed almost no IFNγ release, which we hypothesized as insufficient to support tumor inflammation and downstream endogenous anti-tumor immunity (6, 49). The decoupling ratios (normalized to the benchmark) varied between 2.4 and 11.9 (**Figure 3B**). The intermediate VHH-2-LC and VHH-2-tandem decoupling ratios represent the preferred starting point for further optimization of the TCEs, as they resemble standard second generation decoupled TCEs with apparent but small “cytokine windows” (17, 20), defined as the dose range in which maximal killing is reached but cytokine release may be modulated from very low for safety to nearly that induced by first generation TCEs. As seen for the anti-ROR1 scFv formats, in cases where the second VHH was placed farther apart from the CD3 binding moiety, decoupling capacity increased, which is in line with our findings above. Next, VHH-2-LC was chosen for optimization of the cytokine window, as it displayed better biophysical properties compared to VHH-2-tandem (data not shown).

**Figure 4:**
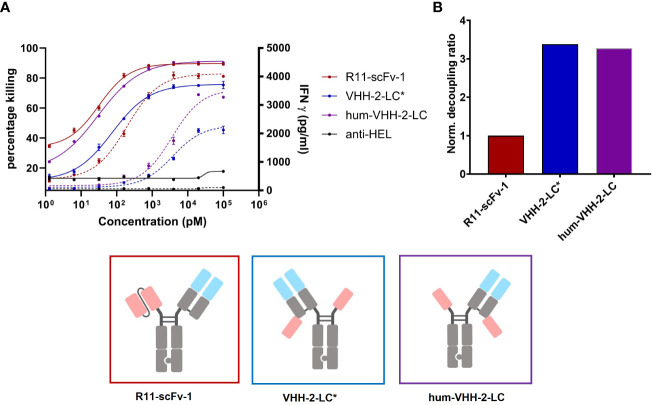
Capacity of the humanized TCE to induce T cell mediated killing and IFNγ release. The TCE hum-VHH-2-LC (purple) was compared to the previously investigated VHH-2-LC (non-humanized) (blue) and the non-decoupled R11-scFv-1 (red). Tumor cell viability and IFNγ release were measured at 24 hours in duplicates. Error bars are based on the SD. Expanded T cells were used as effector cells at a E:T ratio of 3:1. **(A)** Combined x-y plot of tumor cell killing and IFNγ release (dashed line). The humanized hum-VHH-2-LC showed a greater killing potency (EC50) and efficacy (Emax) compared to VHH-2-LC* and was comparable to R11-scFv. Hum-VHH-2-LC had an Emax of 91% and an EC50 of 30 pM while VHH-2-LC* had a significant lower Emax of 76% and an EC50 of 74 pM in killing tumor target cell. For IFNγ release, the potency of hum-VHH-2-LC was 19-fold lower compared to R11-scFv-1. R11-scFv-1 had an Emax of 4,097 pg/mL and an EC50 of 195 pM while hum-VHH-2-LC had a similar Emax of about 3,556 pg/mL but a significantly greater EC50 (lower potency) of about 3.6 nM in inducing IFNγ release. This allows for a concentration range of hum-VHH-2-LC in which maximum tumor cell killing can be achieved without concomitant maximum release of IFNγ termed a cytokine window. **(B)** The corresponding decoupling ratios were shown in a bar graph. R11-scFv-1 was used as the non-decoupled TCE control and reference for normalization. The humanized hum-VHH-2-LC showed similar decoupling capacity as the non-humanized VHH-2-LC. All assays were repeated in at least four and up to eight donors and representative results are shown.

For VHH 5A1, the tasks of humanization and affinity maturation were combined. The former activity allowed for faster development of the TCE and the latter was performed to enhance the cytotoxic properties of VHH-2-LC (using R11-scFv-1 as our benchmark). The resulting humanized version of 5A1 had a higher apparent affinity for ROR1 of ~3 nM (**Supplementarys Figure S1, S3**). The TCE harboring the humanized and affinity matured VHH (hum-VHH-2-LC) was compared with the non-humanized one (VHH-2-LC) and the non-decoupled benchmark, R11-scFv-1 (**Figure 4A**). T cell mediated cytotoxicity increased using the humanized and affinity-matured variant from an EC50 of 74 pM to 30 pM and an Emax of 76% to 91%, which was not appreciably different from R11-scFv-1. However, the amount of IFNγ released remained significantly lower for hum-VHH-2-LC compared to R11-scFv-1 (**Figure 4B**) at the lowest dose at which maximum killing was observed (beginning of the cytokine window but increased to within 10-20% of the benchmark at the end of the cytokine window). Importantly, the cytokine window was large, representing a dose range of approximately 2 logs.

Such a cytokine window is hypothesized to facilitate dosing for maximum killing potency within a range focused on safety (little to no IFNγ release) to that focused on IFNγ’s anti-tumor properties, which can be modulated at both an inter-patient and intra-patient basis depending on tolerability. Importantly, the decoupling ratio did not change appreciably during humanization and affinity maturation.

### Analysis of decoupled hum-VHH-2-LC

**Figure 5:**
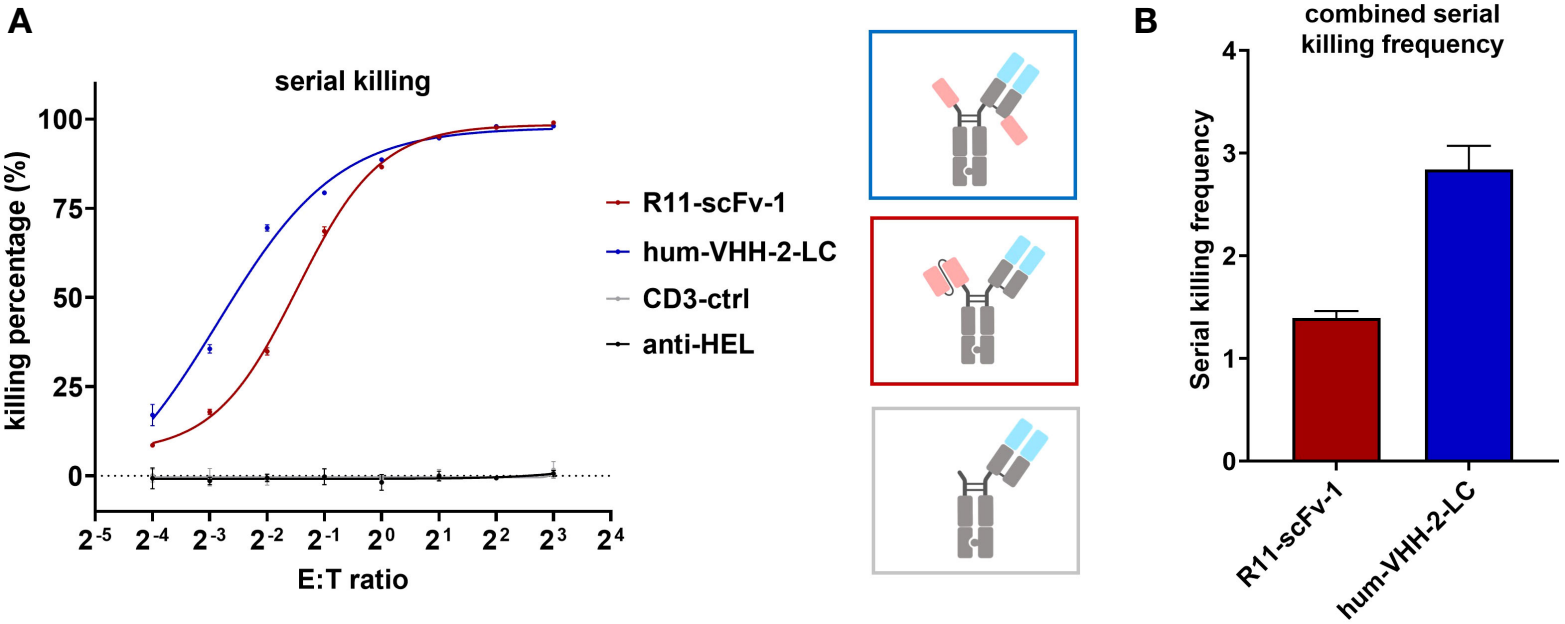
Hum-VHH-2-LC induces superior serial killing relative to the non-decoupled TCE control. **(A)** Analysis of tumor cell viability at different ratios of effector cells (T cells) to ROR1+ MDA-MB-231 tumor cells (E:T ratio) in the presence of 1 nM hum-VHH-2-LC and R11-scFv-1. The number of tumor cells was kept constant (5,000 cells), whereas the number of expanded CD8+ T cells derived from a healthy donor ranged from 300 to 40,000. E:T ratios of 1:16, 1:8, 1:4, 1:2, 1:1, 2:1. 4:1 and 8:1 were analyzed. Tumor cell viability was measured at 24 hours in duplicates. Error bars are based on the SD. CD3-ctrl and anti-HEL were used as negative controls. At E:T ratios of 1:2 to 8:1, no significant difference was observed in tumor cell killing. At lower E:T ratios, hum-VHH-2-LC induced a higher level of T cell mediated ROR1 dependent tumor killing than R11-scFv 1. **(B)** Serial killing comparison of hum-VHH-2-LC and R11-scFv-1. The E:T ratios of 1:16 and 1:8 were combined for the calculation of the serial killing frequency which was defined as the mean number of killed tumor cells by one T cell over a 24 hour period. The decoupled hum-VHH-2-LC supported a greater degree of serial killing at approximately 2.8 tumor cells/T cell than the non-decoupled R11-scFv-1 with about 1.4 tumor cells/T cell. All assays were repeated in at least two donors and representative results are shown.

To further characterize hum-VHH-2-LC, we performed a 24 hour serial killing assay by analyzing T cell mediated tumor killing at very low E:T (effector: tumor cell) ratios at which killing frequency above a known threshold would necessitate multiple kills by the same T cell. For this purpose, activated CD8+ T cells were used. At higher E:T ratios (>1:2), no significant difference in tumor cell killing was observed between R11-scFv-1 and hum-VHH-2-LC (**Figure 5**). Such an E:T ratio may reflect the environment in hematologic tumors. At very low E:T ratios (1:16 and 1:8), more representative of solid tumors, we observed that serial killing associated with hum-VHH-2-LC was 2.8 tumor cells per T cell. In comparison, R11-scFv-1 induced negligible serial killing of only 1.4 tumor cells per T cell. Our observation suggests that hum-VHH-2-LC is a superior therapeutic for solid tumors, where the number of T cells may be sparse with low E:T ratios (50).

**Figure 6:**
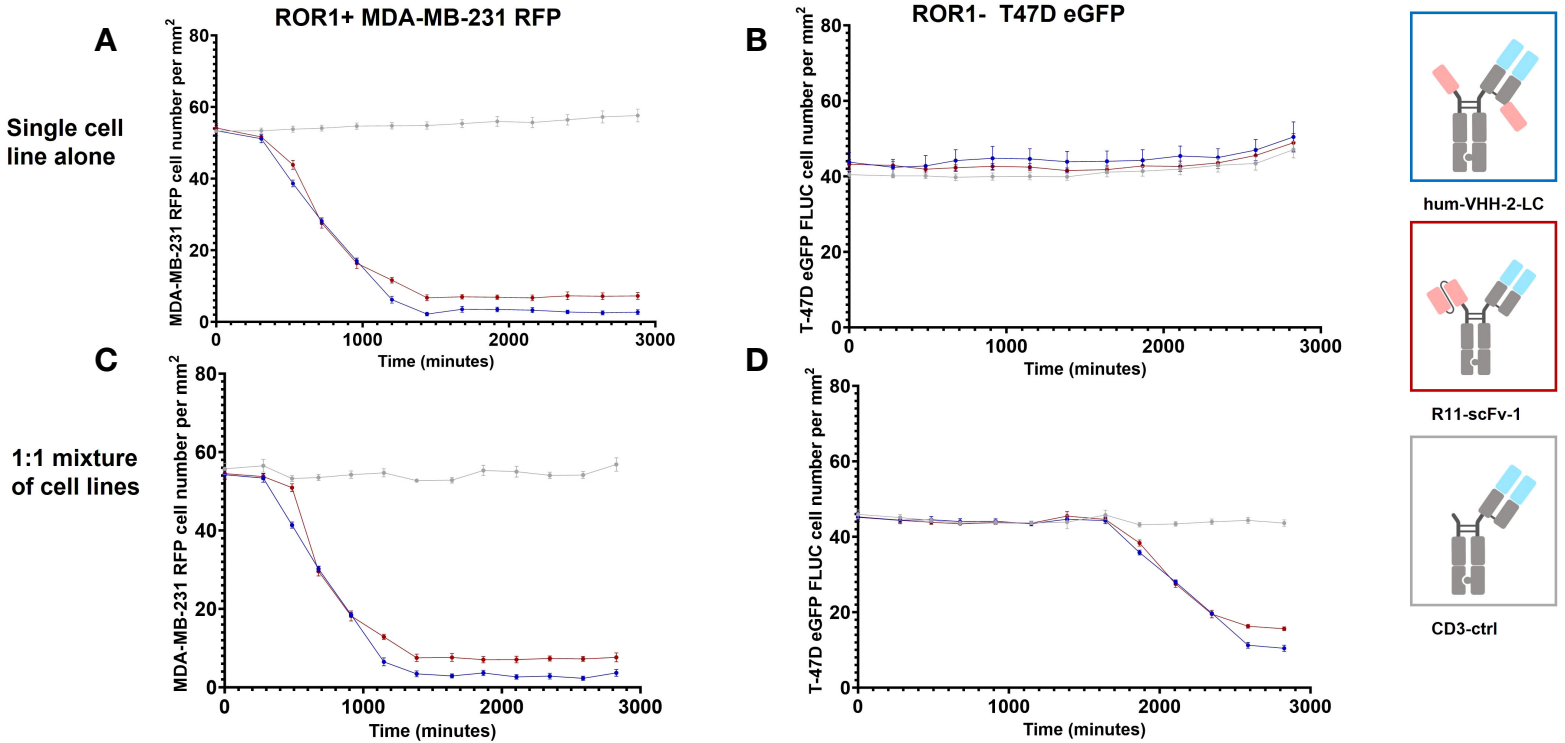
Kinetic analysis of TCE induced bystander killing of ROR1 negative tumor cells in a heterogenous mixture of tumor cells. Kinetic measurements were performed at a construct concentration of 1 nM. **(A)** Hum-VHH-2-LC induced T cell mediated killing of about 95% of ROR1+ MDA-MB-231 tumor cells with an IC50VIABILITY of about 13.3 hours. In comparison, T cells killed approximately 87% of ROR1+ MDA-MB-231 tumor cells in the presence of 1 nM R11-scFv-1. Expanded T cells were used as effector cells at an E:T ratio of 5:1. **(B)** When ROR1- T-47D tumor cells were co-cultured with expanded T cells, no killing of tumor cells was observed in the presence of TCEs. **(C)** When expanded T cells were used as effector cells in co-culture with a 1:1 mixture of ROR1+ MDA-MB-231 and ROR1- T-47D tumor cells, similar killing of ROR1+ tumor cells was observed in comparison to the cultivation with the ROR1+ cell line alone but at a later timepoint. A ratio of 5:1:1 was used for T cell, ROR1+ MDA-MB-231 cell and ROR1 T-47D cell. **(D)** In the co-culture of ROR1+ and ROR1- tumor cells, ROR1- tumor cells were killed in the presence of 1 nM hum-VHH-2-LC and R11-scFv-1. After 47.1 hours hum-VHH-2-LC induced T cell mediated killing of about 77% of ROR1- T-47D tumor cells, whereas R11-scFv-1 induced killing of 66%. The IC50VIABILITY in the presence of hum-VHH-2-LC was about 35.1 hours and 34.2 hours in the case of R11-scFv. In all assays the negative control CD3-ctrl and anti-HEL showed no cell killing. Measurements were made in duplicate wells using four read areas per well. Error bars are based on the SD. All assays were repeated in at least two donors and representative results are shown.

**Figure 6** represents the results of our assessment of bystander killing for hum-VHH-2-LC and the benchmark. Bystander killing is defined as the killing of TAA negative (ROR1 in our case) tumor cells in the presence of ROR1 positive tumors and is therefore an important component of killing tumors expressing heterogeneous levels of a TAA, a very common occurrence in the solid tumor setting. Bystander killing has been reported to be induced by death receptors (e.g. FAS) in cooperation with adhesion molecules (e.g. ICAM-1) and that the upregulation of both is dependent on IFNγ (6). Therefore, the question arises whether the decoupled hum-VHH-2-LC induces sufficient IFNγ release for bystander killing. As such, we examined the kinetics by which hum-VHH-2-LC and the benchmark R11-scFv-1 induced expanded T cell mediated killing of ROR1-positive MDA-MB-231, ROR1-negative T-47D tumor cells or a 1:1 mixture of both cells. This was performed at multiple TCE concentrations within hum-VHH-2-LC’s cytokine window. Shown in **Figure 6** is one exemplary concentration (1nM), representing a concentration only about 10% into the cytokine window. A dose response within the cytokine window was observed (**Supplementary Figure S4**). The results revealed that hum-VHH-2-LC was as efficient at inducing direct killing of ROR1-positive MDA-MB-231 and bystander killing of ROR1-negative T-47D as R11-scFv-1. This suggests that the cytokine window associated with hum-VHH-2-LC can support IFNγ dependent killing of heterogenous tumors. The kinetics for killing ROR1-positive MDA-MB-231 when co-cultured with ROR1-negative T-47D cells was similar to that observed with MDA-MB-231 alone. Such early direct killing of the TAA+ target has been previously shown to be perforin/granzyme mediated (6). In contrast, bystander killing of ROR1-negative T-47D only occurred when co-mixed with MDA-MB-231 and was delayed by about 35 hours (measured using the delta in IC50_VIABILITY_ between the ROR1+ and ROR1- tumor cells in the mixture). The observation is consistent with a previous report describing cytokine dependent bystander killing induced by a BiTE targeting EGFR (6).

**Figure 7:**
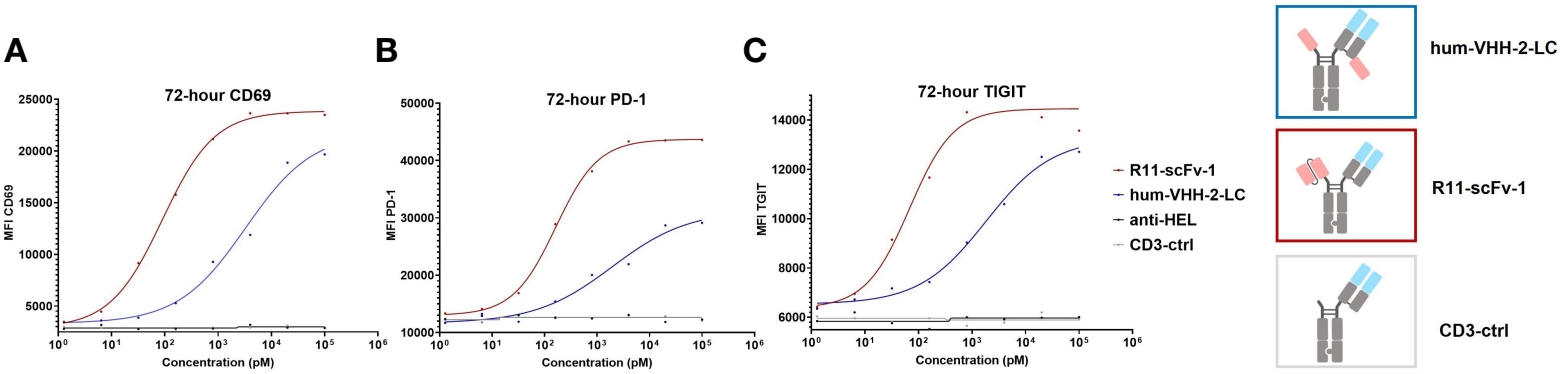
TCE-induced expression of CD69, PD-1 and TIGIT on T cells. The upregulation of cell surface CD69, PD-1 and TIGIT expression on T cells was measured by mixing PBMCs with ROR1+ MDA-MB-231 cells at the ratio of 30:1. After the addition of TCEs (decoupled hum-VHH-2-LC and the non-decoupled R11-scFv-1) in duplicates, CD69, PD-1 and TIGIT were measured at 72 hours by flow cytometry. CD3-ctrl and anti-HEL were utilized as negative controls. **(A)** For CD69 surface expression, hum-VHH-2-LC showed an EC50 of about 2 nM, whereas R11-scFv had an EC50 of 163 pM. **(B)** For PD-1 expression, hum-VHH-2-LC had a lower EC50 of about 3.3 nM in comparison to R11-scFv-1 (90 pM). **(C)** The induction of TIGIT surface expression was also less potent for hum-VHH-2-LC (1.8 nM) compared to R11-scFv-1 (68 pM). All assays were repeated in at least two donors and representative results are shown.

We also sought to investigate the impact of hum-VHH-2-LC and the benchmark R11-scFv-1 on the induction of markers of activation and exhaustion on T cells upon co-incubation with ROR1+ tumor cells. To do so, the upregulation of T cell surface expression of CD69, PD-1 and TIGIT (51–54) within PBMC was analyzed when co-mixed with ROR1-positive MDA-MB-231 tumor cells (**Figure 7**). The results demonstrated that that hum-VHH-2-LC induced appreciably less upregulation of cell surface expression of CD69, PD-1 and TIGIT on T cells compared to R11-scFv-1. Using EC50 as our readout for the propensity of an “exhaustive phenotype”, we found that in comparison to R11-scFv-1, hum-VHH-2-LC was about 12-fold, 36-fold, and 27-fold less prone to inducing the upregulation of CD69, PD-1 and TIGIT, respectively. The results indicate that, within hum-vHH-2-LC’s cytokine window, this TCE is appreciably less likely to induce T cell exhaustion relative to the first generation benchmark TCE and therefore maintains preferred T cell fitness.

**Figure 8:**
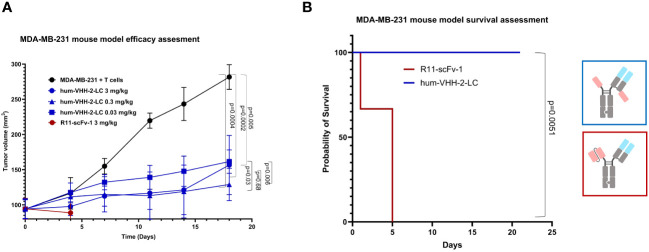
Tumor growth inhibition in a mouse tumor xenograft model. NSG mice were subcutaneously implanted with the human ROR1+ TNBC cell line, MDA-MB-231, and intravenously reconstituted with expanded human T cells from a healthy human donor. When tumor volume reached about 100 mm3, mice were grouped and received one of PBS (5 mice), 3mg/kg hum-VHH-2-LC (10 mice), 0.3 mg/kg hum-VHH-2-LC (10 mice), 0.03 mg/kg hum-VHH-2-LC (10 mice) or 3mg/kg R11-scFv-1 (7 mice). Administration was performed intraperitoneally twice a week. **(A)** Tumor volume was displayed as a function of time. For all dose levels of hum-VHH-2-LC tested, a statistically significant TGI was observed (p values ranging from 0.005 to 0.0002). Mice treated with R11-scFv-1 died within the first five days after the first administration. **(B)** Mice treated with all dose levels of hum-VHH-2-LC survived for 21 days when the study was terminated, whereas all mice treated with R11-scFv-1 died within five days.

Lastly, we investigated the capacity of hum-VHH-2-LC to mediate tumor growth inhibition (TGI) in a mouse xenograft model (**Figure 8**). NSG mice were subcutaneously implanted with human ROR1-positive MDA-MB-231 cells and intravenously reconstituted with expanded human T cells. Although the overall growth of the tumors was slow due to apparent alloreactivity of the transplanted T cells towards the tumor, we were able to observe statistically significant TGI from hum-VHH-2-LC at all dose levels tested (0.03, 0.3 and 3 mg/kg; p values ranging from 0.005 to 0.0002). No significant difference in TGI was observed between the two higher dose levels. We did, however, note that the lowest dose level was associated with reduced TGI relative to the higher doses (p = 0.006 - 0.03) and thus suggestive of a dose-response that plateaued at 0.3 mg/kg. Interestingly, cell surface ROR1 expression on tumors excised from mice at termination was observed to drop from an average of 82% in mice treated with PBS to ~42% and ~26% in mice treated at 0.3 mg/kg and 3 mg/kg, respectively (data not shown; tumors from mice treated at 0.03 mg/kg were not examined), suggestive of a further dose response between the two higher doses. This difference in presumed anti-tumor activity between the two higher doses was likely difficult to assess via tumor volume alone due to the overall slow tumor growth rate. The study was terminated at 21 days because of retarded overall tumor growth interpreted as resulting from donor T cell mediated alloreactivity and consistent with T cell reconstitution reaching as high as 45% of mouse peripheral blood at this time (data not shown). Graft versus tumor mediated growth retardation is common to the MDA-MB-231 xenograft model in PMBC reconstituted mice, impacting 2 out of 3 donors (55), and nonetheless yields results acceptable in the field (56). Notably, all mice treated with hum-VHH-2-LC survived for 21 days without weight loss or behavior related morbidities, while all mice treated with the non-decoupled reference R11-scFv-1 died within the first 5 days after the first treatment from toxicity associated with splenomegaly and hepatomegaly, both of which have been reported as manifestations of CRS (57).

## Discussion

T cell engagers (TCEs) have emerged as a powerful immunotherapeutic, especially for hematologic tumors but adverse events including CRS and on-target-off-tumor toxicity as well as the poor fitness of T cells in solid tumors have limited their utility in the latter space (1, 58). Here, we have reported the engineering of a ROR1 specific TCE that decouples cytotoxicity from cytokine release, has a wide cytokine window and is associated with improved T cell fitness relative to even second generation TCEs. ROR1 was chosen because of its expression on a wide range of tumors in both the solid and liquid spaces. This latter attribute partially derisks a potential asset for clinical development. To make the TCE highly modular, we used nanobodies generated in *Camelus bactrianu*s specific for the distal Ig domain. VHHs are characterized by good expressibility and an increased paratope diversity. The latter is the case due to structural peculiarities such as an additional disulfide bridge between the CDR loops (47). This new class of TCEs are associated with a cytokine window. This is defined as a dose range in which maximum killing is observed but cytokine release can be modulated from very little for safety to that postulated to induce robust tumor inflammation (designated here as the maximum cytokine release induced by our first generation TCE benchmark). The cytokine window is associated with a right shifted EC50 relative to that for cytotoxicity, which is thought to support not only a larger therapeutic window relative to first generation TCEs but also bystander killing, shown by Ross and colleague to require IFNγ (6).

Our approach for engineering a TCE with a cytokine window was to first identify a TCE geometry that supported decoupling of cytotoxicity from cytokine release via modulation of apparent synaptic range and then tailor the apparent affinity of ROR1 specific arms to create a more stable synapse. A stable synapse was reported by Cremasco et al. as required for generating rapid and persistent T cell/tumor conjugates linked to optimal *in-vivo* anti-tumor activity (49). The authors observed that the IFNγ-CXCL10γ axis is a secondary dependent factor for optimal activity. This was linked to tumor resident T cell proliferation and recruitment of peripheral T cells into the TME, both of which are maintained in a controlled fashion within the cytokine window.

The VHH-based construct that met the decoupling criterium was VHH-2-LC. Importantly, VHH-2-LC induced significantly lower IFNγ release in comparison to the previously described R11-based TCE (42). VHH-2 was humanized to decrease the risk of immunogenicity and affinity matured to increase potency and optimize the cytokine window. The TCE harboring the humanized VHH, hum-VHH-2-LC, showed a comparable decoupling ratio to the non-humanized parent but improved T cell mediated killing comparable to R11-scFv-1 and a cytokine window that ranged from the release of about 15% to 90% that of R11-scFv-1. Hum-VHH-2-LC showed improved serial killing in comparison to the non-decoupled R11-scFv-1. Moreover, hum-2-VHH-LC was able to induce bystander killing similar to R11-scFV-1, which directly indicates the capacity of hum-VHH-2-LC to target tumors expressing heterogeneous levels of ROR1 and indirectly suggests that hum-VHH-2-LC can promote infiltration of immune cells into tumors and endogenous and durable tumor specific immunity. Hum-VHH-2-LC also induced less upregulation of the T cell exhaustion markers CD69, PD-1, and TIGIT (51–54) in comparison to the reference R11-scFv-1 suggesting that hum-VHH-2-LC may be further associated with improved T cell fitness. To our knowledge, hum-VHH-2-LC is the first TCE reported to decouple cytotoxicity not only from TIGIT and PD-1 but also the early activation marker, CD69. This observation suggests that release of cytolytic granules can be decoupled from the expression of CD69. It provides further support of decoupling from exhaustion because CD69 has also been reported to constitute a functional marker of early exhaustion (51).

Importantly, our *in-vitro* observations were corroborated *in-vivo* such that tumor bearing humanized NSG mice treated with a high dose (3 mg/kg) of the non-decoupled R11-scFv-1 died from multi-organ failure including hepatomegaly, often oberserved as associated with CRS (57). Unlike our 5A1 VHH that does not bind mouse ROR1, the R11 paratope recognizes both human and mouse ROR1 (**Supplementary Figure S5**) and can be used to assess on-target-off-tumor toxicity in mice. Interestingly, R11 CAR-T (43) but not R11xCD3 TCE analogous to our R11-scFv-1 (42), had previously been reported to cause lethality in mice (43). Such lethality was linked to the lymphodepletive preconditioning regimen used prior to adoptive transfer, resulting in insufficient hematopoietic reconstitution post-transplant. Hematopoietic pathology was attributed to upregulation of ROR1 in bone marrow osteoblasts and mesenchymal stem cells. No signs or symptoms of CRS were reported (43). Furthermore, no observation of bone marrow pathology was observed in mice treated with R11 CAR-T or R11 TCE in the absence of a lymphodepletive preconditioning regimen (42, 43). In our hands and in contrast to treatment with high dose R11-scFv-1, treatment with high dose hum-VHH-2-LC resulted in strong efficacy and no signs of weight loss or behavior morbidities in the observed timeframe. This finding exemplifies the potential therapeutic value of a decoupled TCE with a cytokine window.

Others have also generated TCEs in which cytotoxicity is decoupled from cytokine release but these TCEs are largely only associated with reduced maximum IFNγ release with little right shifting of their EC50s relative to cytotoxicity. Examples of such TCEs are as follows (1) Zuch de Zafra et al. modulated the affinity of both the CD3 and TAA targeting arms of a of a 1 + 1 CD38 specific TCE (25, 2), Hernandez-Hoyos et al., generated a PSMA Adaptir with mid affinity (low double digit nM range) binding arms. This TCE was especially interesting because of its nearly complete decoupling of cytotoxicity from cytokine release, which likely resulted from the combination of a membrane distal epitope selection on PSMA and the relatively large apparent synaptic range mediated by an Adaptir, in which the CD3 binders are located on the opposite side of an Fc scaffold relative to the TAA binders (59, 3). Two further TCEs (PSMA and BCMA specific, utilizing a novel CD3 binding fab, clone F2B, have been reported. The authors suggest that F2B binds a unique epitope on CD3δϵ (with low affinity) that is intrinsically linked with not only decoupling of cytotoxicity from cytokine release but also selective activation of cytotoxic versus regulatory T cells, the latter of which representing a feature we could not duplicate. Close inspection of F2B indicates that it is in fact a moderate affinity CD3 complex binder (low double-digit nM) with extremely low maximum binding capacity. While it is possible that it’s binding site on the CD3 complex impacts apparent synaptic range, both CD3δ and CD3ϵ are small proteins of approximately 35 kDa suggesting that (a) the detuned nature of F2B containing TCEs please change to: and (b) the binding site on the TAA are responsible for the reduced magnitude of cytokine release induced by these TCEs as opposed to the unique nature of F2B’s epitope (20, 23).

The mechanism by which modulation of the T cell-tumor apparent synaptic range results in a cytokine window remains to be fully elucidated. We hypothesize the following model: Hum-VHH-2-LC generates an apparent synaptic range similar to that observed by Li and colleagues with a FcRH5 specific TCE, whereby CD45 is partially excluded from the synapse (29). Unlike the FcRH5 TCE, however, hum-VHH-2-LC is able to mediate cytotoxicity as strong as the non-decoupled benchmark because we strengthened the synapse and increased its persistence by affinity maturing the ROR1 targeting VHH. This is consistent with the finding of Ameen Al-Aghbar et al. who investigated the spatial distribution of CD45 and the TCR signaling apparatus within synapses formed between T cells and glass beads coated with either a high or low affinity anti-CD3 scFv derived from clone OKT3 fused to either a small linker or a large CD43 scaffold. As expected, CD45 was present in the synapse generated by the low affinity scFv fused to CD43. Interestingly, however, substitution for the high affinity scFv not only resulted in partial exclusion of CD45 and moderate phosphorylation of Zap70 but also T cell proliferation of similar magnitude to that induced by the short scFv that fully excluded CD45 (60). Our model of a “tight” synapse combined with a relatively long apparent synaptic range mediating generation of a cytokine window can help explain why affinity modulation alone can only decouple by reducing the magnitude of cytokine release. In this scenario, the low affinity binders for CD3 and/or the TAA generate a weak synapse that allows for CD45 to continuously go into and out of. This is consistent with the finding of Faroudi et al. who found that low avidity interactions between CMV specific T cells and CMV peptide pulsed antigen presenting cells were associated not only with decoupling of cytotoxicity from cytokine release but also an undulating pattern of T cell activity that the authors termed “calcium flux spikiness” (31). Interestingly, the modest but appreciable cytokine window observed in the CMV specific T cell model suggests that the low affinity/high avidity interactions characteristic of TCR/MHC-I-peptide recognition may be an important variable to consider. Indeed, it will be interesting to investigate carefully whether the 2 + 1 and higher order avidity formats of TCEs provide an advantage over 1 + 1 formats. Not all formats, however, benefit from valency. For example, Bacac et al. employed a 2 + 1 crossfab format in which one of two moderate affinity TAA binders (low double digit nM range) was fused directly to the CD3 binder. Although steric hindrance between the TAA specific fab and the hinge domain of the Fc scaffold resulted very low apparent affinity for CD3, the TCE did not decouple cytotoxicity from cytokine release presumedly because the synaptic distance was too short (61).

Perhaps the greatest challenge is predicting the synaptic distance that results in decoupling of cytotoxicity from cytokine release. Given the multiple factors involved in decoupling and the variable size of a cytokine window generated, it is likely that the best insight possible will be a range of distances. Given that the ectodomain of CD45 ranges in size from ~28 nm (CD45RO) to ~50 nm (CD45ABC) (62, 63), we already have a starting point. Indeed, using the mouse ovalbumin specific T cell hybridoma B3Z and CHO cells expressing its MHC-I/peptide ligand attached to scaffolds of differing lengths, Choudhuri et all reported that an intermembrane distance of ~28 nm largely reduces their capacity to measure B3Z mediated IL-2 release (34). IL-2 is consumed by T cells so it is possible the aforementioned assay system underestimated the distance to fully abrogate activation of B3Z. Interestingly, we do have insight into the approximate distance that can convert a non-decoupled 2 + 1 TCE to a decoupled one. We observed that when targeting the ROR1 Ig domain with the 5A1 VHH, that VHH-VHH-2-LC and VHH-2-HC but not VHH-2-Fab decoupled cytotoxicity from cytokine release (see formats in **Figure 3**, **Supplementary Figure S6**). Using the crystal structures of exemplary IgG1 (PDB ID: 1hzh) and VHH (PDB ID: 1fvc) and an estimated average length for each amino acid in a flexible linker as 0.3-0.4 nm, we calculated the distance between the CDR3 region of the anti-CD3 Fab on the N-terminal knob to the CDR3 region of the 5A1 VHH that was not fixed on the N-terminal hole. These lengths equated to 22.6 nm (VHH-2-HC), 12.2 nm (VHH-2-LC), and 10 nm (VHH-2-Fab) suggesting that an increased distance between the CD3 and ROR1 binding arms of 2-12 nm may be sufficient to convert a non-decoupled 2 + 1 TCE into a decoupled TCE. Our data does not exclude the possibility that format may unintentionally impact the apparent affinity for CD3 and/or the TAA. However, distance between the binding arms appeared to be a key variable because the degree of decoupling trended with the distance between the binding arms such that VHH-2-HC (22.6 nm) and VHH-2-LC (12.2 nm) were ~6 fold and 2.5 fold decoupled relative to VHH-2-Fab (10 nm). In addition to our findings, Chen et al. observed that a BCMA targeted IgG2 based TCE (9-15 nm) could decouple cytotoxicity from cytokine release but the same binders in diabody format (3-6 nm) could not (26). Their calculated delta between IgG2 and diabody (6-9 nm) is consistent with the distance we calculated above (2-10 nm) as likely important for converting a ROR1 Ig domain specific non-decoupled TCE into a decoupled TCE. As such, although measuring synaptic distance (T cell/tumor intermembrane distance) was beyond the scope of this work and hence termed “apparent synaptic range”, we predict that a distance shorter than the ectodomain of CD45 that facilitates a cytokine window could be as little as the deltas described above but would vary depending on the CD45 isoform(s) expressed and other proteins, especially adhesion molecules, co-localized in the same synapse. The rigidity and glycosylation of different CD45 isoforms is likely a further complicating factor. In a recent report by Staufer and colleagues, the authors provide insight into the impact of the TCE-determined intermembrane distance of a functional T cell/tumor synapse on the potency of the TCE. They concluded that a potent TCE mediates first the adhesion, then CD45 exclusion and co-stimulation recruitment, which are dependent on the spacing between the CD3 and TAA binding sites and the flexibility of the TCE. The authors found that single digit nm differences in the distance between TCE binding arms in a 1:1 configuration had an appreciable impact on T cell activity that arose mostly from altering the “level of forming close contacts and inside-out signaling” (30). Of note, it was reported that T-EMRA cells make up the bulk of cytotoxic T cells in the TME of established solid tumors so perhaps the size and rigidity of CD45RA’s ectodomain is most physiologically relevant in this context (32, 64). It should be noted that our distance model is rather speculative and further experiments such as measurement of 1:1 constructs and also cryoEM and microscopic studies are needed to gain more insight into the mechanistic principles.

In summary, by generating an array of 2 + 1 TCE formats, we were able to achieve a broad range of decoupling ratios which could be optimized for a wide cytokine window by stabilization of the T cell/tumor synapse via affinity maturation of the TAA binder. Generation of such a cytokine window was the key finding of this work and we acknowledge that the complex nature of TCE induced T cell/tumor synapses discussed above necessitates further investigation to propose a mechanistic model. The lead ROR1 TCE identified, hum-VHH-2-LC, served as a case study for combined modulation of TCE mediated apparent synaptic range and bridge strength and may have potential as an impactful therapeutic for both solid and liquid tumors.

## Data availability statement

The raw data supporting the conclusions of this article will be made available by the authors, without undue reservation.

## Ethics statement

Ethical approval was not required for the studies on humans in accordance with the local legislation and institutional requirements because only commercially available established cell lines were used. The animal study was approved by a CRO called Innomodels according to their AUCUC. The study was conducted in accordance with the local legislation and institutional requirements.

## Author contributions

XZ: Data curation, Investigation, Methodology, Writing – review & editing. FG: Investigation, Writing – original draft. DH: Writing – review & editing, Supervision. JT: Conceptualization, Writing – review & editing, Project administration. HK: Conceptualization, Project administration, Writing – original draft. BR: Conceptualization, Writing – original draft, Project administration.
