# Supplementary material for: Using protein geometry to optimize cytotoxicity and the cytokine window of a ROR1 specific T cell engager
Source: Front Immunol. 2024 Feb 22;15:1323049. doi: 10.3389/fimmu.2024.1323049 (PMC10917902; doi:10.3389/fimmu.2024.1323049)
Supplement: Supplementary file 1 [file DataSheet_1.pdf]

Supplementary Material

Using protein geometry to optimize cytotoxicity and the cytokine window of a ROR1 specific T cell engager

Xueyuan Zhou[1+], Felix Klaus Geyer[2+], Dominic Happel[2], Jeffrey Takimoto[1], Harald Kolmar[2,3*], Brian Rabinovich[1*]

[1]Fuse Biotherapeutics, Woburn, MA, USA

[2]Institute for Organic Chemistry and Biochemistry, Technical University of Darmstadt, Peter-Grünberg-Strasse 4, D-64287 Darmstadt, Germany

[3]Centre for Synthetic Biology, Technical University of Darmstadt, Darmstadt, Germany

*** Correspondence:**
Brian Rabinovich

brabinovich@fusebiotx.com

Harald Kolmar

Harald.Kolmar@TU-Darmstadt.de

[+] These authors have contributed equally to this work and share first authorship.

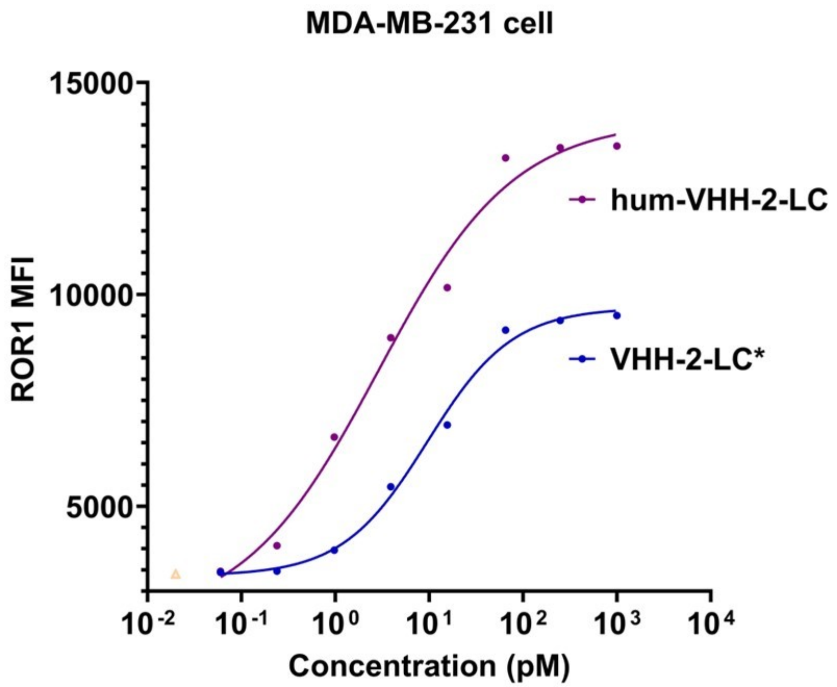

Supplementary figure S1: Apparent affinity determination of 5A1 and humanized 5A1 by flow cytometry. Shown is a non-linear x-y plot of the magnitude of binding to ROR1 on MDA-MB-231 (MFI) of hum-VHH-2-LC (purple closed circles; EC50 ~3 nM) and VHH-2-LC (blue closed circles; EC50 ~20 nM) as a function of test article concentration.

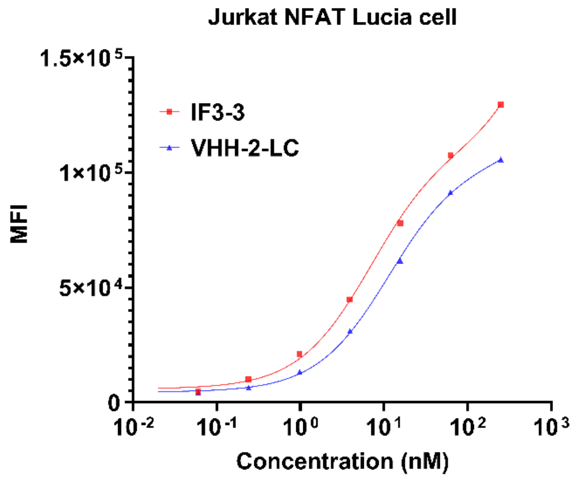

Supplementary Figure S2. Apparent affinity determination of CD3 binder IF3-3. Binding to Jurkat NFAT Lucia cells was analyzed. For IF3-3 alone an EC50 value of 6.9 nM was determined. For VHH-2-LC consisting of one IF3-3 moiety it was 11.4 nM.

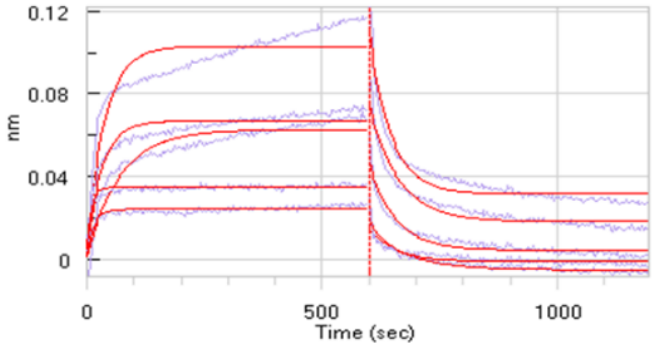

Supplementary figure S3: Binding kinetics of monovalent hum_VHH 5A1 to human ROR1 was examined by BLI measurement. The calculated K_D is approximately 110 nM. The kinetic parameters are $k_{on} = 4.35*10^4\ M^{-1}s^{-1}$ and $k_{off} = 5.02*10^{-3}\ s^{-1}$.

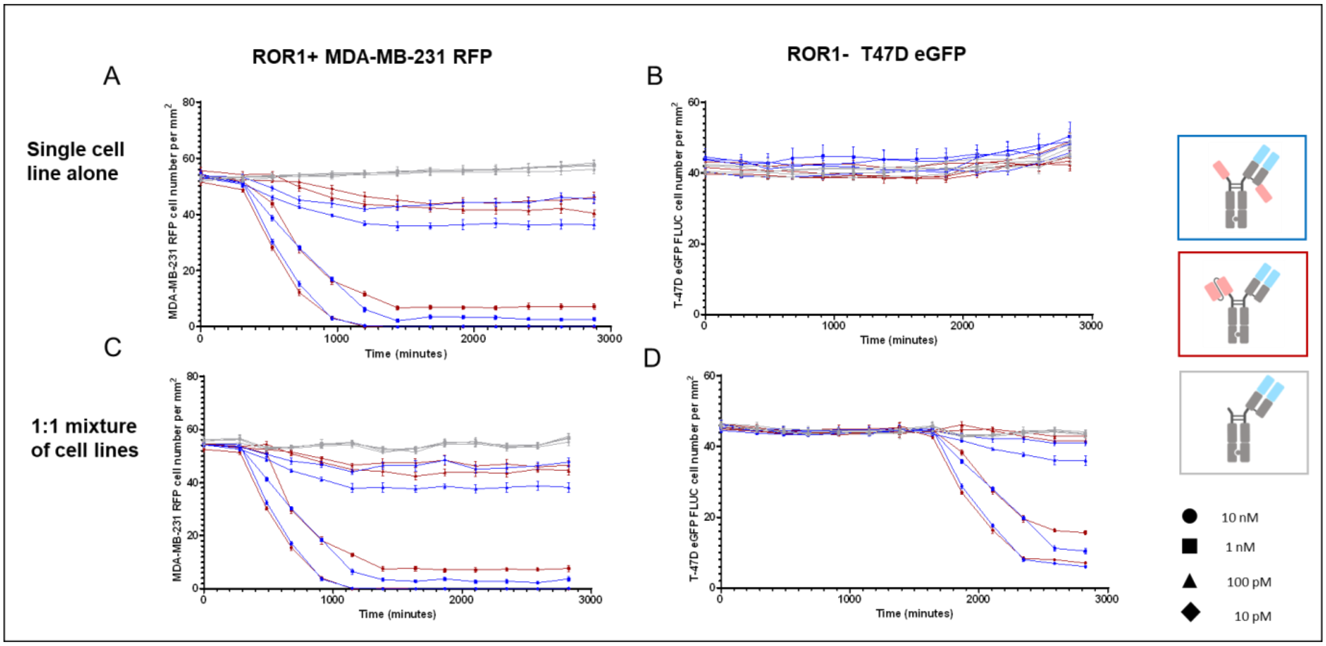

Supplementary Figure S4. Kinetic analysis of TCE induced bystander killing of ROR1 negative tumor cells in a heterogenous cell mixture at different concentrations of TCEs. Kinetic measurements were performed at a construct concentration of 10 nM, 1 nM, 100 pM, and 10 pM. Expanded T cells were used as effector cells at an E:T ratio of 5:1. A Similar concentration-dependent killing of ROR1 positive was observed for R11-scFv-1 and hum-VHH-2-LC. B When ROR1- T-47D tumor cells were co-cultured with expanded T cells, no killing of tumor cell was observed with the presence of TCEs. C When expanded T cells were used as effector cells in co-culture with a 1:1 mixture of ROR1+ MDA-MB-231 and ROR1- T-47D tumor cells, similar killing of ROR1+ tumor cells was observed in comparison to the cultivation with the ROR1+ cell line alone. A ratio of 5:1:1 was used for T cell, ROR1+ MDA-MB-231 cell and ROR1 T-47D cell. D In the co-culture of ROR1+ and ROR1- tumor cells, ROR1- tumor cells were killed in a concentration-dependent manner in the presence of hum-

VHH-2-LC and R11-scFv-1In all assays the negative control CD3-ctrl showed no cell killing. Measurements were made in duplicate wells and four read areas per well.

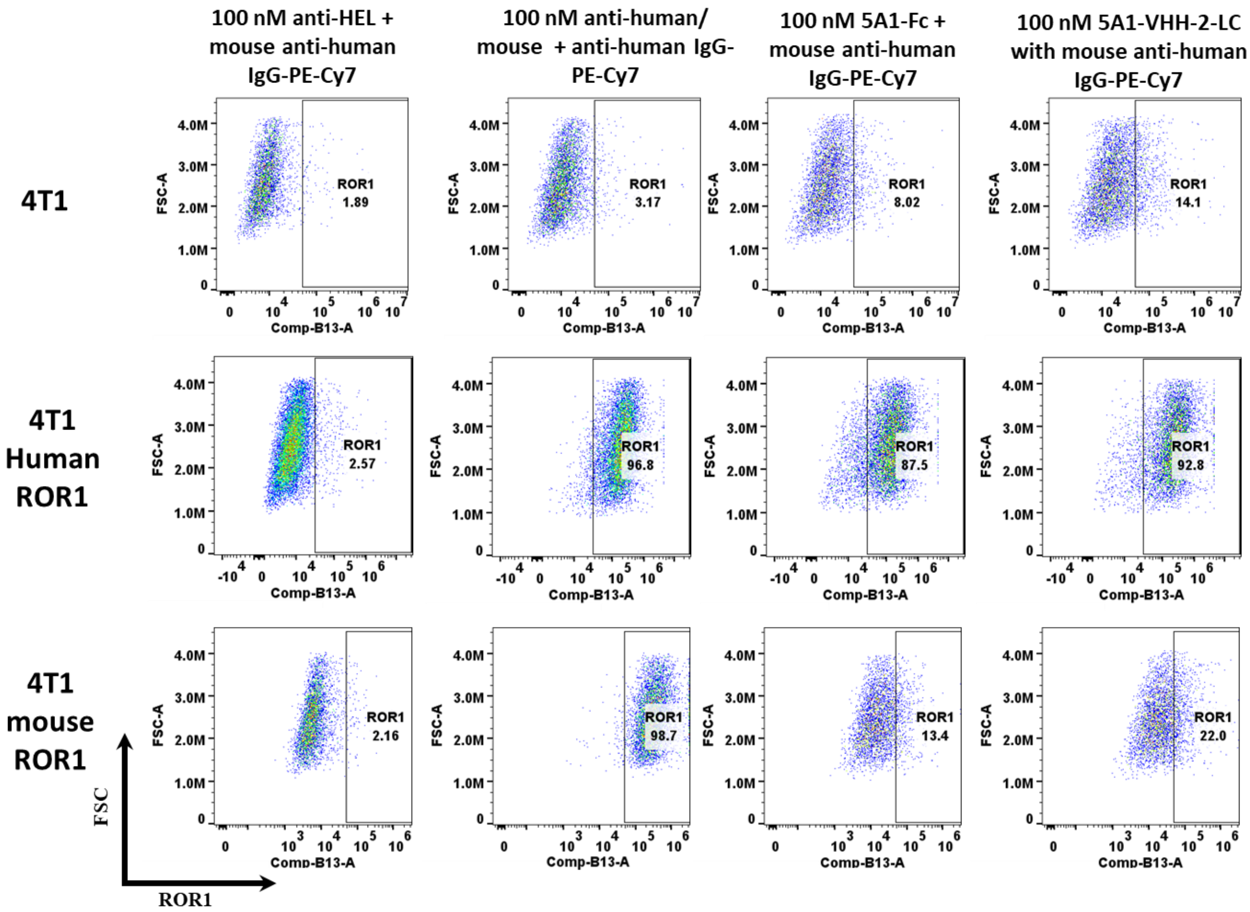

Supplementary figure S5: Cytometric flow analysis showed binding of the VHH 5A1 to 4T1 cells engineered to express human ROR1 but not to those expressing mouse ROR1.

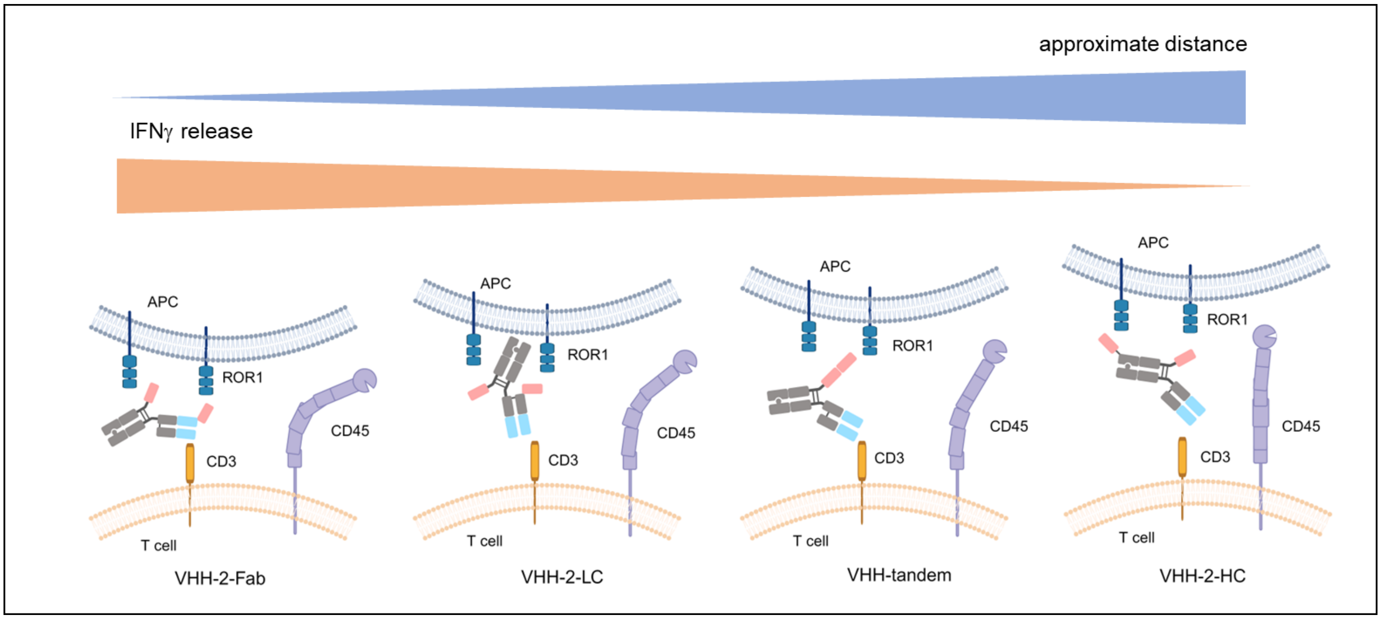

Supplementary Figure S6: Illustrative figure displaying interaction of different bispecific antibody formats with target ROR1 on APC and CD3 on T cell.
